# Acute thoracolumbar pain due to cholecystitis: a case study

**DOI:** 10.1186/s12998-015-0079-2

**Published:** 2015-12-18

**Authors:** Chris T. Carter

**Affiliations:** School of Health Professions, Murdoch University, 90 South Street, Murdoch, WA 6150 Australia

## Abstract

**Background:**

This article describes and discusses the case of an adult female with cholecystitis characterized on initial presentation as acute thoracolumbar pain.

**Case Presentation:**

A 34-year-old female presented for care with a complaint of acute right sided lower thoracic and upper lumbar pain with associated significant hyperalgesia and muscular hypertonicity. The patient was examined, referred, and later diagnosed by use of ultrasound imaging.

**Conclusion:**

Despite many initial physical examination findings of musculoskeletal dysfunction, this case demonstrates the significance of visceral referred pain, viscerosomatic hyperalgesia & hypertonicity, and how these neurological processes can mimic mechanical pain syndromes. A clinical neurological discussion of cholecystitis visceral pain and referred viscerosomatic phenomena is included.

## Background

Chiropractors are trained as primary contact providers and well positioned to provide initial assessment, diagnosis and treatment for patients with spinal pain in addition to assisting in referral to other practitioners [1]. The presentation of back pain is common to chiropractors and differentiating acute musculoskeletal (somatic) from visceral pain presentation may be difficult as they can present with a similar clinical pain picture. These challenging clinical presentations can lead to inappropriate and delayed patient care, serving as a reminder of the importance of performing a thorough history and physical examination.

Visceral pathology as a presenting complaint to chiropractors is generally considered rare, however a survey of American chiropractors indicated that 5.3 % of primary complaint presentations are non-musculoskeletal in origin [2]. It is estimated that up to 15 % of the American population have gallstones, 10-18 % of whom will develop biliary pain, and 7 % of which will require operative intervention [3]. With over 700,000 cholecystectomies performed annually in America, gallbladder disease is considered the most costly digestive disorder [3].

Although the majority of those with cholelithiasis will not develop acute cholecystitis, 1-3 % inevitably will. The transition of acute cholecystitis leading to a secondary bacterial infection of the gallbladder may occur in up to 50 % of cases. This increases the chance of complications such as the formation of an empyema, perforation, widespread peritonitis, sepsis and abdominal abscesses [4, 5]. Earlier intervention of acute cholecystitis has been increasingly recognized in a recent meta-analysis study that demonstrated performing early laparoscopic cholecystectomy (within 1–7 days of symptom onset) decreased incidence of complications including wound infections, provided shorter length of hospital stay and decreased costs versus delayed cholecystectomy. No difference in rates of mortality were noted. The authors advocated that early laparoscopic cholecystectomy is now considered best care and should be considered a routine in patients presenting with acute cholecystitis [6].

The statistics above provide evidence why early detection of acute cholecystitis by a primary contact practitioner may reduce risk of complications and help reduce costs. Unfortunately, there is no single clinical symptom or sign that carries sufficient weight to establish or exclude cholecystitis without right upper quadrant ultrasound testing [7]. Traditional cholecystitis symptoms (right upper quadrant pain, nausea, and emesis) and signs (Murphy, right upper quadrant tenderness and fever) have poor positive and negative likelihood ratios [7]. Further research into combining presenting signs and symptoms is needed as currently the use of clinical gestalt continues to be considered the most appropriate initial clinical management prior to obtaining more valid blood and imaging diagnostic tests [7].

When back pain is the predominant or only clinical symptom of acute cholecystitis presentation, this may present a challenge to the practitioner. Firstly, being aware that cholecystitis visceral and referred pain can be perceived by patients in a variety of anatomical areas is important [8]. Pain may be perceived in such areas as the epigastrium, right hypogastrium, and lower areas of the right thoracic spine and posterior ribs [3, 9]. Pain perceived in the epigastrium and right hypogastrium may be true visceral pain, however pain felt in the back is referred viscerosomatic pain [8].

Distinguishing spine pain from somatic or visceral origin initially relies on a thorough history and physical examination. Pain of visceral origin tends to be characterised as sharp, cramping or achy, and is diffuse, poorly localized and perceived deep inside the body [9]. It usually starts insidiously, not relieved by rest or sleep, and may improve temporarily with short-term activity [10]. The physical examination for assessing cholecystitis will not only include checking for fever, abdominal tenderness and presence of Murphy sign, but should be aimed at using physical body movements and orthopaedic provocative tests to see if the back pain can be reproduced, worsened or relieved . This may help distinguish between visceral (no significant change with movement) and somatic back pain. Somatic pain is typically worsened or relieved with particularly range of motion movements or with provocative testing that stretch and/or compress somatic tissue [11].

The presence of somatic tissue hyperalgesia and muscle hypertonicity is a known common finding in somatic mechanical disorders of the spine [12]. However, cholecystitis referred pain to somatic structures also commonly presents with hyperalgesia and muscle hypertonicity in the somatic referred area [13]. This can make a more difficult differentiation between somatic and visceral pain during the physical examination as the patients back pain can theoretically be worsened with physical movements or palpation of somatic tissue in both presentations.

A thorough history should also inquire information on patient positive risk factor(s) for cholelithiasis. This is helpful in increasing the clinician’s awareness that a pain presentation may be visceral in origin, and would be considered a part of the practitioner’s clinical gestalt. Cholelithiasis development is multifactorial, however common risk factors include age over twenty years, female sex, history of pregnancy, parity, obesity, and ironically any recent rapid weight loss [3].

This case study describes an adult female presenting with cholecystitis characterized by acute right sided thoracolumbar pain. It highlights the challenges faced when visceral pathology creates viscerosomatic symptoms and signs that present similar to mechanical musculoskeletal clinical presentation. Prompt referral to the appropriate medical health care provider is necessary to limit possible patient health complications and may improve management. A detailed clinical neurological discussion of cholecystitis visceral and referred pain, as well as the neurological phenomena of viscerosomatic hyperalgesia and muscular hypertonicity is included to assist the clinician in understanding how visceral pathology can mimic mechanical musculoskeletal syndromes.

## Case presentation

### Clinical history and examination

A 34 year-old female presented with two days of insidious onset right sided lower thoracic and upper lumbar pain. The pain was constant, worse with movement, and particularly increased with deep inspiration and laughing. No abdominal or limb pain, paraesthesia or numbness was noted. Her numerical rating scale (NRS) was 6/10, and she mentioned the pain felt slightly different yet no more severe than several previous episodes of lower lumbar pain. Characteristically, her pain was achy and deep, with no associated symptoms of nausea, malaise, fever, bloating, emesis, dysuria or haematuria. It was no worse or better around meals or alcohol intake. The patient was not in distress however appeared slightly more anxious as usually her back pain was not so constant.

The patient had presented several times over the preceding two years for chiropractic care for episodes of mechanical lower lumbar, neck, right hip and left shoulder pain. Her prior chiropractic treatments had consisted of spinal manipulation, mobilisation, myofascial release, rehabilitation and education. The patient’s musculoskeletal complaints usually recovered successfully with conservative management. Static mechanical allodynia had been consistently noted axially and peripherally over the previous two years, however no other symptoms or signs indicating fibromyalgia diagnosis were noted. In fact, the patient’s allodynia had been improving steadily in congruence with her increase in exercise over the preceding couple of years.

The youngest of her three children was two years old, and she had been increasing her frequency, duration and intensity of exercise over the preceding 1.5 years with regular jogging, boot-camp, and crossfit classes. Her increased exercise had resulted in reduction of weight from 92 kg to 85 kg, as well as a significant gain in strength and sense of well-being. Initial fear-avoidance behaviour to exercise post-partum due to chronic right hip pain of gluteal medius tendinopathy origin had taken several months to improve, and included education and graded progression of exercise.

Medical health history was significant for left sided Erb’s palsy at parturition, and resection of 85 % of her pancreas at age five weeks due to intractable neonatal hypoglycemia. She had gestational diabetes during all three pregnancies and had current type 2 diabetes. No current medications were being consumed. Family history was unknown.

Physical examination revealed unremarkable lumbar neurological examination and lumbar nerve root stress tests. Active lumbar and thoracic range of motion caused increased right-sided back pain in all ranges along the T8-L2 segments in the area of the erector spinae muscles. Active and passive right lumbar lateral flexion and right lumbar Kemp’s test particularly worsened her back pain. Using the Modified Ashworth Scale for grading hypertonia, this patient had Grade 2 muscle hypertonicity noted with palpation of the right sided lower thoracic and upper lumbar erector spinae and psoas muscles in contrast to Grade 0 on the left side. Palpation underneath the right costal margin elicited only mild tenderness without sharp pain or inspiratory arrest (i.e., negative Murphy sign).

### Diagnosis and management

There were obvious signs of musculoskeletal involvement to her acute thoracolumbar back pain. However, due to the new, more constant pain presentation and positive cholecystitis risk factors in this patient that included pain location, female sex, age over twenty, parity (3 children), patient habitus, and significant weight-loss over the prior year, there was high suspicion of acute cholecystitis. Initial presentation was a weekend day and she was recommended and referred by letter to see her general practitioner on the following Monday for clinical opinion. She was also recommended to go to the hospital immediately if her pain worsened, began to affect the abdominopelvic region, or if she felt unwell. The patient consented to a trial treatment of myofascial release and spinal manipulation in hope that her symptoms and signs may just be of mechanical origin and some pain relief may be achieved. The patient felt slightly better immediately post treatment however interestingly pre- and post-treatment muscle hypertonicity was palpably unchanged.

Three days post initial presentation she had a diagnostic ultrasound which revealed clinical signs of acute cholecystitis including focal tenderness over the gallbladder, a 25 mm non-mobile stone at the body/fundal region, a moderate amount of sludge extending towards the gallbladder neck, slight thickening of the gallbladder wall, and no evidence of pericholecystic fluid. Phone discussion with the patient the day of the ultrasound revealed that she had recently developed right hypogastric pain which was severe post ingestion of fatty foods. Additionally, her back pain had increased in intensity to a NRS of 8/10.

She was diagnosed with acute cholecystitis by her General Practitioner, was prescribed antibiotics and paracetamol and referred to a specialist. Within a week of medication introduction and restriction of high fatty meal intake, her back and abdominal pain had significantly decreased to a NRS of 1/10. Fifty-three days after initial presentation she had a successful laparoscopic cholecystectomy with no complications. Monthly follow-up over the next six months with this patient post-surgery revealed no current back or abdominal pain in the previously affected thoracolumbar area. She returned to exercise two month’s post-surgery.

In this case, delayed cholecystectomy was decided by her specialist. Therefore, early referral to her general practitioner likely did not affect her temporal surgical outcome. However, early referral assisted in early diagnosis via ultrasound which may have reduced secondary bacterial complications and improved patient comfortability as she was prescribed antibiotics and paracetamol.

## Discussion

### Visceral pain of gallbladder origin

The majority of acute cholecystitis cases are due to the presence of cholelithiasis [5, 14]. The production of gallstones and sludge at or near the neck of the gallbladder can cause obstruction of bile into the cystic duct, causing a number of pathophysiological nociception contributors that result in the production of visceral pain [5]. Once obstructed, the continued production of gallbladder mucus has no outlet for drainage and causes distension of the gallbladder wall and ducts [15]. The subsequent increased peristalsis causing strong and sustained smooth muscle contraction results in increased intraluminal pressure. This activation of gallbladder nociceptors by distension can occur in the absence of visceral inflammation and may present clinically as episodic biliary colic [14, 16].

Biliary system nociception has two distinct types of peptidergic bare nerve-ending afferent sensory fibres that respond to noxious stimulation located in the mucosa, muscle and serosa [17, 18]. Two-thirds of these fibres have low-thresholds to biliary pressure and encode stimuli in both the innocuous and noxious range in addition to transmitting information related to autonomic phenomena. One-third respond only to high-threshold biliary stimuli in the noxious range and are functionally similar to cutaneous nociceptors [15, 19].

Initial painful contraction of the gallbladder appears to involve high-threshold receptors, whereas ischemia-induced hypoxic and inflammatory tissue environments that damage the mucosa peripherally sensitize both high and low threshold as well as visceral silent nociceptors [4, 19, 20]. Gallbladder nociceptors also release substance P and vasoactive intestinal peptide to induce a neurogenic inflammatory environment which appears to be a prominent contributor to cholecystitis development [21]. Prostaglandins, in addition to their peripheral sensitization effects, also increase afferent nociceptive signalling by stimulating wall contraction and secretion of lumen fluid resulting in wall thickening, pericholecystic oedema, and peritonitis [4] Interestingly, the severity of visceral pathology is not well correlated with the severity of pain perceived, which attributes attention to unknown peripheral mechanisms as well as the role of spinal and higher brain centre mechanisms of pain perception [22].

Nociceptor afferents from the gallbladder are carried anatomically via vagal and splanchnic nerves, however the majority of nociception travels through the latter with their central terminations in dorsal and ventral horns of the spinal cord extending from T5 to L2 [11, 15, 22]. Therefore, the anatomical area of visceral pain perceived in acute cholecystitis significantly varies depending on which spinal segment second order neurons are stimulated over such a broad spinal cord distribution.

Visceral pain is characterised as sharp, cramping or achy, although tends to be diffuse, poorly localized and perceived deep inside [9]. This is due to the relative paucity of peripheral afferent nociceptor terminals in the viscera, central terminal arborisation, distinct absence of a separate visceral sensory pathway, and overall low proportion of visceral afferent nociceptors compared with those of somatic origin [19, 22]. The reality that such a low number of arriving visceral afferent signals can result in such a profound pain with possible additional autonomic phenomena (for example malaise and nausea) is likely due to the extensive arborisation and resultant functional divergence seen in the cord as well as the stimulation of so many cord levels at once [23].

Visceral nociceptor central terminals synapse with second-order neurons in lamina I, II, V, and X of the spinal cord [22]. Visceral nociception then projects to higher centres via several routes that lead to perceived pain as well as autonomic phenomena commonly associated with visceral pain. Stimulation of the spinomesencephalic, spinoreticular, and spinohypothalamic tracts mainly activate unconscious and/or automatic responses to visceral sensory input including alterations in behaviour and emotion [17]. The major pain perception pathways are the contralateral spinothalamic tract and more recently understood ipsilateral dorsal fasciculus that both project via nuclei of the thalamus to higher brain centres such as the somatosensory, anterior cingulate, prefrontal and insular cortices [17, 18]. The unique addition of strong emotional, behavioural and autonomic features commonly associated with heterogenous visceral pain presentation is likely due to the widespread distribution of afferent pathways to areas beyond those required for localisation alone [17]. Pre-existing affective and cognitive dysfunction may also play a role in acute visceral pain perception.

### Gallbladder referred pain

Referred pain is commonly perceived in cholecystitis and can be an important clinical diagnostic measure [24]. The occurrence of referred pain is best explained by the convergence-projection model which postulates that the majority of dorsal horn second-order neurons that receive afferent nociceptive input from viscera also happen to receive input from somatic structures that share the same neuromeric field [13, 24]. In cholecystitis, referred pain to the back is explained by the dual synapsing of gallbladder afferents on the same viscerosomatic second-order neurons in the spinal cord that also receive innervation from somatic afferents coming from the right lower thoracic spine and ribs [16]. The convergence-projection model also involves overlapping visceral and somatic nociception signalling to similar higher brain centres involved in the perception of pain, however the brain likely attributes the origin of the sensation to the somatic domain because of pneumonic traces of previously experienced somatic pain [22].

### Hyperalgesia and muscular hypertonicity

Cholecystitis referred pain to somatic structures commonly presents with hyperalgesia and muscle hypertonicity in the somatic referred area [13]. This hyperalgesia is likely due to ‘convergence-facilitation’ which is caused by neuroplastic central sensitization changes in the central nervous system due to the massive barrage of visceral afferent impulses from an algogenic inflamed and mechanically irritated gallbladder [13, 25]. Acute cholecystitis and biliary calculosis has been shown to cause mechanical and thermal hyperalgesia in superficial structures such as the subcutaneous tissue and skin, as well as efferent sympathetically induced trophic changes [24]. This can present as a diagnostic challenge when assessing patients such as this female who had shown previous long-term signs of static mechanical allodynia. Central sensitization hyperalgesic mechanisms may be involved in continual pain post cholecystectomy, however clinically this uncommonly occurs if adequate treatment is not delayed [26].

Muscle hypertonicity is a common finding in many mechanical musculoskeletal disorders. However, strong evidence exists for reflex efferent induced muscle contraction as a cause of the hyperalgesia seen in referred visceral pain syndromes [25]. Muscle contraction is commonly seen clinically in patients with acute visceral pain, and has been histologically shown in rats to involve significant microscopic changes in muscle fibre phenotype [27]. Referred pain to somatic structures is likely to involve not just central changes but also peripheral changes such as altered neurotransmitters and sensory terminals in the affected somatic tissue that affect tissue contractility [25].

### Modulation of visceral pain

No discussion in pain presentation is complete without briefly mentioning the complex modulation that occurs in an individual’s pain perception and the subsequent variable pain presentations that can ensue even in the acute patient. Similar to somatic pain, modulation of true and referred visceral pain occurs at both the spinal cord and the brain [25]. For example, in between biliary colic episodes, the nature of decreased perceived pain may be due to increased spinal cord neuron thresholds due to transient inhibition of transmission to higher centres [17]. Depending on the nature of the visceral stimulus, descending pathways from supraspinal structures can inhibit or facilitate spinal viscerosomatic neurons in both the dorsal and ventral horn [23].

The emotional state of individuals also appears to have a significant modulatory influence on visceral pain [17]. The final conscious and subconscious psychological processing and therefore perception of visceral pain in the higher pain matrix centres shares similarities but differs from that of somatic pain processing [23]. Visceral nociception likely plays a significant role in acute visceral pain perception, however the psychological influences of cognitions, mood and social setting are also likely to play an important role in pain perception and should never be under-appreciated.

## Conclusion

Relatively uncommon in daily chiropractic practice, gallbladder pathology mimicking mechanical musculoskeletal back pain does occur and clinicians should be aware of its presentation due to it being one of the most frequent medically encountered gastrointestinal presentations. Similar clinical symptoms and signs may arise due to the anatomical areas of pain involved as well as the neurophysiological mechanisms of viscerosomatic hyperalgesia and muscular hypertonicity that commonly occur with biliary pain presentations. Timely differentiation of clinical pain is helpful in undertaking prompt referral to assist the patient in receiving appropriate medical care which may reduce complications and include advantageous early cholecystectomy.

## Consent

Written informed consent was obtained from the patient for publication of this case report. A copy of the written consent is available for review by the Editor-in-Chief of this journal.
